# Association between dietary inflammatory index and polycystic ovary syndrome: a meta-analysis of case-control studies

**DOI:** 10.3389/fendo.2026.1843644

**Published:** 2026-08-18

**Authors:** Junmei Huang, Chunyan Dong, Danyi Qiu, Xiaoye Li

**Affiliations:** 1 Department of Nutrition, Sanya Central Hospital (The Third People’s Hospital of Hainan Province), Sanya, China; 2 Reproductive Medicine Department, Sanya Central Hospital (The Third People’s Hospital of Hainan Province), Sanya, China

**Keywords:** case-control study, dietary inflammatory index, meta-analysis, polycystic ovary syndrome, pro-inflammatory diet

## Abstract

**Objective:**

This study aimed to systematically analyze the association of the dietary inflammatory index (DII) with polycystic ovary syndrome (PCOS) status, thus offering evidence-based support for dietary interventions in the management of PCOS.

**Methods:**

Four databases were systematically searched to collect case-control studies on DII and PCOS published up to December 21, 2025. The Newcastle-Ottawa Scale (NOS) was utilized to assess the risk of bias in the included studies. Using Stata 15.0, we conducted a meta-analysis to compare DII scores in PCOS patients versus those in healthy controls. Subgroup analyses were conducted to identify sources of heterogeneity. An exploratory meta-regression was conducted, but due to the small number of studies, the results could not be interpreted.

**Results:**

Six case-control studies involving 1,554 participants were ultimately included. Meta-analysis revealed slightly but statistically significantly higher DII scores in the PCOS group than in healthy controls (standardized mean difference [SMD]: 0.23; 95% confidence interval [CI]: 0.06–0.40; P = 0.008). Sensitivity analyses suggested robustness of the results. Egger’s test showed no statistical evidence of publication bias (P = 0.611); however, with only six studies the statistical power was very low, so publication bias cannot be excluded, and this analysis should be regarded as exploratory.

**Conclusion:**

Pro-inflammatory dietary patterns are modestly associated with PCOS. Although causality inference is impossible due to the design of included studies, existing evidence supports DII as a potential tool for estimating PCOS status associated with diet. The small effect size observed in this study indicates a significant association between DII and PCOS. However, statistical association does not necessarily imply practical clinical relevance, and further studies are warranted to replicate and validate this relationship.

**Systematic Review Registration:**

https://www.crd.york.ac.uk/PROSPERO/view/1239576, identifier CRD420251239576.

## 1 Introduction

Polycystic ovary syndrome (PCOS) represents an endocrine-metabolic disorder commonly detected in women of childbearing potential, affecting approximately 6% to 20% of women worldwide (1). It manifests as a diverse range of symptoms, encompassing reproductive dysfunction such as oligomenorrhea, ovulatory disorders, hyperandrogenism, and polycystic morphology of the ovaries. Moreover, patients with PCOS frequently experience elevated risks of chronic metabolic conditions, such as impaired sensitivity to insulin, type 2 diabetes, obesity, abnormal lipid profile, and cardiovascular pathology. PCOS imposes a substantial burden on women’s health throughout their lifespan (2). Although the precise pathogenesis of PCOS remains insufficiently understood, a multifactorial pathogenesis model involving interactions between genetic susceptibility and environmental and lifestyle factors has been widely recognized. Chronic inflammation, acting as a bridge linking metabolic abnormalities and reproductive dysfunction, has become a hotspot of recent research on the pathogenesis of PCOS (3). Among the numerous environmental and lifestyle factors that can modulate inflammatory states, dietary patterns are considered the most promising and actionable intervention targets (4).

Various nutrients and food components in daily diets can directly or indirectly influence the body’s oxidative stress levels and inflammatory responses through complex metabolic and signaling pathways (5). In 2009, researchers from Columbia University first proposed the Dietary Inflammatory Index (DII) based on a global investigation conducted between 1950 and 2007 examining the link between dietary patterns and inflammatory indices (6). By scoring dietary intake data, this index comprehensively evaluates whether an individual’s long-term dietary pattern is theoretically pro-inflammatory or anti-inflammatory. This approach provides a standardized scoring system designed to evaluate the total inflammatory burden potentially attributed to diet patterns (7). The introduction of DII not only addresses the limitations of single-nutrient analysis but also offers a novel perspective for the prevention of chronic diseases by integrating the intricate association linking dietary patterns with inflammatory responses (8). Multiple observational studies published in recent years have identified an evident link between high DII and a greater likelihood of developing PCOS. For instance, a case-control study involving 44 PCOS patients and 44 healthy participants has shown significantly higher DII scores in PCOS patients than in healthy participants (9). Another study involving Middle Eastern participants has further confirmed that the Dietary and Lifestyle Inflammation Score (DLIS) outperforms DII alone in predicting PCOS, suggesting that insufficient physical activity and high body mass index (BMI) may amplify the harmful effects of pro-inflammatory diets (10). A mechanistic study has indicated that pro-inflammatory diets induce systemic inflammatory responses by increasing intestinal permeability, promoting the translocation of endotoxins into the bloodstream, and activating TLR4 receptors, which subsequently disrupt ovarian follicular development and hormone synthesis, as reported in a preprint (11).

Given the close theoretical link of diet to inflammation and PCOS, the association of DII with PCOS status has become a recent research hotspot in nutritional epidemiology. Several case-control studies have explored this association, but their conclusions remain inconsistent. For instance, a 2023 case-control study has indicated that elevated energy-adjusted DII (E-DII) scores, indicating dietary patterns with greater pro-inflammatory potential, are positively correlated with PCOS status (12). A 2022 review has explicitly noted that pro-inflammatory diets may contribute to reproductive disorders, including PCOS (13). However, another study has suggested that this association may be influenced by multiple factors such as race, geographic region, obesity status, and PCOS phenotype, leading to inconsistent effect sizes and even contradictory conclusions (14). Furthermore, all current evidence originates from observational studies, predominantly case-control studies. Thus far, no systematic analysis has specifically associated DII with PCOS.

Given this, this meta-analysis aimed to comprehensively synthesize data from observational studies exploring the association between DII and PCOS status. Through rigorous literature screening, quality assessment, and data synthesis, our study sought to clarify whether a highly pro-inflammatory diet (a high DII score) is associated with the stuatus of PCOS. The study is expected to provide evidence-based support for elucidating diet-related risk factors for PCOS. Meanwhile, it offers a scientific reference for developing PCOS prevention and management strategies centered on anti-inflammatory diets, holding significant value for clinical practice and public health.

## 2 Methods

### 2.1 Registration and MOOSE statement

This study strictly adhered to the Meta-analysis Of Observational Studies in Epidemiology (MOOSE) guidelines (15) during the study design, implementation, and report writing to ensure transparency of the study process and completeness of the reporting (**Supplementary Table 1**). The study protocol was pre-registered on December 4, 2025 in the International Prospective Register of Systematic Reviews (PROSPERO) (CRD420251239576) to facilitate the assessment of consistency between the study protocol and the final report, thereby minimizing the risk of bias due to selective reporting.

### 2.2 Literature search

Two independent researchers comprehensively searched PubMed, Embase, Cochrane Library, and Web of Science databases from their inception to December 21, 2025. Additionally, to minimize publication bias and avoid the omission of relevant studies, reference lists of selected articles were manually screened, and relevant clinical trial registries were searched for potential unpublished gray literature. The search strategy involved combinations of subject headings and free-text terms and was tailored to each database. Key search terms included “polycystic ovary syndrome,” “dietary inflammatory index,” and their corresponding synonyms. During study selection, any discrepancies between the two reviewers were addressed through discussion or by reaching a consensus with a third reviewer. The complete search strategy, including MeSH terms, Boolean operators, and the filters applied to each database, is detailed in **Supplementary Table 2**.

#### 2.2.1 Inclusion and exclusion criteria

Inclusion criteria comprised: (i) Study Population: Female patients with PCOS meeting the international diagnostic criteria who were of reproductive age; (ii) Study type: Case-control studies; (iii) Outcome measure: Since all included studies employed a case-control design, the primary outcome measure of this meta-analysis was the standardized mean difference (SMD) in DII scores between the two groups, which reflects cross-sectional differences between groups rather than a prospective prediction of the risk of developing the disease.

Exclusion criteria comprised: (i) Case reports, animal studies, reviews, commentaries, letters, and conference abstracts; (ii) Studies enrolling pregnant or lactating women, subjects with other major diseases that may significantly affect endocrine or inflammatory markers, or subjects with recent use of medications potentially influencing the study outcomes; (iii) Studies failing to quantitatively assess dietary patterns, or evaluating a single nutrient only, or using other diet scores unrelated to inflammation as primary exposure factors; (iv) Studies with unavailable full text or insufficient data for extraction and synthesis, studies failing to report core outcome measures, or studies with non-convertible or unextractable effect sizes; (v) Duplicate publications, articles with only abstract and no full text, and non-English-language articles.

#### 2.2.2 Literature screening and data extraction

Two researchers screened articles and extracted data independently. Retrieved studies were screened using EndNote 21. After all records were imported, duplicate records were first removed. All potentially eligible studies were then independently screened through a title and abstract review. Studies clearly irrelevant according to the established criteria were excluded. Articles with full texts were subsequently reviewed for final assessment. Any discrepancies were addressed through discussion or, if needed, by inviting a third researcher for arbitration.

Data were extracted into a pre-designed standardized spreadsheet in Excel. After eligible studies were identified and included, data were extracted from each included study. During data extraction, when consensus could not be reached between the two researchers, a third researcher participated in the discussion to resolve discrepancies. Extracted data comprised: (i) General information of studies: first author, publication year, country; (ii) Study characteristics: sample size, control design, diagnostic criteria, outcome measures; (iii) Characteristics of study subjects: age, BMI, physical activities; (iv) Outcome data: mean DII and corresponding standard deviation. All studies included in the analysis reported DII as the mean ± standard deviation; therefore, no conversion from the median/interquartile range or standard error was necessary. All data analyzed in this study were extracted from previously published articles, which are cited in the reference list.

### 2.3 Risk of bias assessment

The internationally recognized Newcastle-Ottawa Scale (NOS) was utilized to assess the quality of eligible case-control studies (16, 17). The NOS covers three dimensions: selection of cases and controls (4 points), comparability between groups (2 points), and ascertainment and measurement of exposure factors (3 points), with a maximum total score of 9 points. Studies were stratified according to their total scores into high (≥ 7), moderate (5-6), or low (≤ 4 points) quality. The assessment was performed by two independent researchers who received training on the consistent use of the scale prior to the assessment. Discrepancies in scoring were addressed through discussion or arbitrated by a third researcher.

### 2.4 Statistical analysis

All statistical analyses were performed in Stata MP 15.0. For continuous outcome variables, the standardized mean difference (SMD) and its 95% confidence interval (CI) were calculated as effect size measures. The thresholds for the interpretation of SMD values were as follows: minimum (< 0.2), slight (0.2–0.49), moderate (0.5–0.79), and substantial (≥ 0.8).

Heterogeneity was assessed using the Q statistic and I^2^ statistic. Given the anticipated clinical and methodological variability among the included observational studies (e.g., different populations, dietary assessment methods, and PCOS phenotypes), a random-effects model was adopted as the default method for pooling effect estimates. This method accounts for both within-study and between-study variance. None of the included studies had missing data that required imputation, and all studies reported mean DII scores and standard deviations for both the case group and the control group. Subgroup analyses were performed to identify potential sources of heterogeneity. To explore potential sources of heterogeneity, we conducted univariate meta-regression analyses using study-level covariates (including mean age and publication year). Due to the small number of included studies, all meta-regression analyses should be considered exploratory and hypothesis-generating, rather than confirmatory. Therefore, the results should be interpreted with caution, and no causal inferences should be drawn from them. Additionally, sensitivity analysis was performed to examine the stability of our findings. To detect potential publication bias, funnel plots and Egger’s test were employed. P < 0.05 indicated statistical significance. An exploratory meta-regression was conducted, but due to the small number of studies, the results could not be interpreted.

## 3 Results

### 3.1 Literature search results

**Figure 1:**
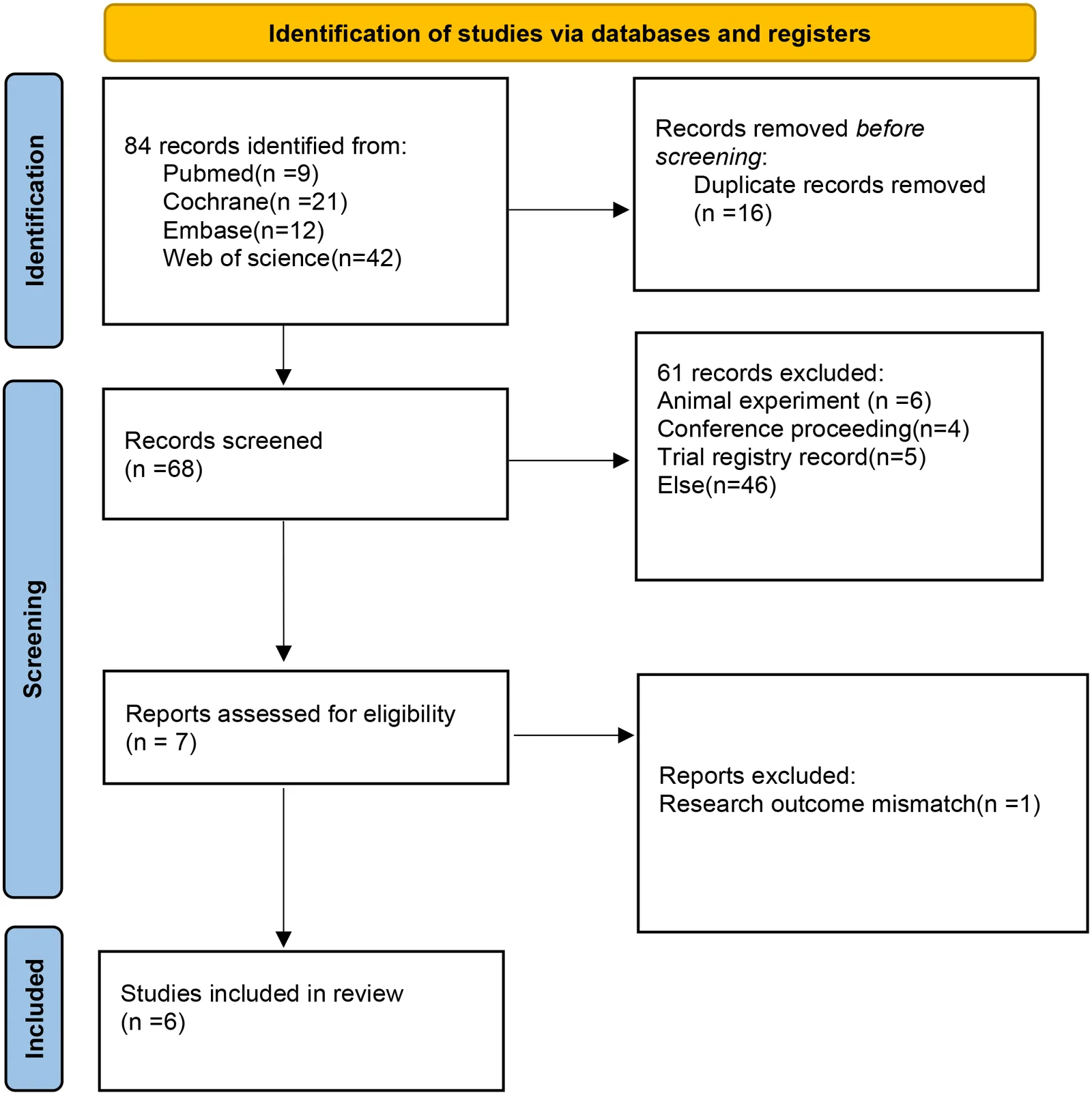
Study screening flowchart.

A preliminary search of the aforementioned four English-language databases yielded a total of 84 articles. After removing 16 duplicates, 68 articles remained. Following screening, 61 were excluded, including 6 animal studies, 4 conference abstracts, 5 trial registration records, and 46 irrelevant articles. The remaining 7 articles underwent full-text evaluation. Among them, one was excluded due to ineligible outcome measures. Ultimately, 6 case-control studies were included (**Figure 1**).

### 3.2 Study characteristics

The basic characteristics of the 6 included studies are summarized in **Table 1**. Among these studies, four studies (10, 18–20) were conducted in Iran, one (21) in China, and one (9) in Turkey. Therefore, all six studies were conducted in Asian populations, with the majority from Middle Eastern countries. Two studies by the same team (Sharkesh et al., 2024 (12) and Sharkesh et al., 2022 (20)) had exactly the same study population. In accordance with predefined exclusion criteria, only the earlier publication (20) was included, while (12) was excluded; since (12) explored EDIP rather than DII, it is cited only conceptually in the discussion. They were published between 2021 and 2025. These studies recruited a total of 1,554 participants, with sample sizes varying between 40 and 325. Their mean age varied from 25 to 32 years. All studies employed the Rotterdam criteria to diagnose PCOS. Quality assessment utilizing the NOS scale indicated that all 6 studies were rated high-quality, as detailed in **Supplementary Table 3**.

**Table 1:** Characteristics of studies included in the meta-analysis.

NO	General information			Demographic information of study population										
	First author	Publication year	Country	Study design	Sample size		Age		BMI		Physical activity (MET-hours/week)		Diagnostic criteria	Outcome indicators
					PCOS	Healthy	PCOS	Healthy	PCOS	Healthy	PCOS	Healthy		
1	Azarbayjani K et al. (19)	2024	Iran	Retrospective case-control study	45	40	27.93 ± 6.64	33.02 ± 7.19	33.02 ± 7.19	25.85 ± 5.07	/	/	Rotterdam Standard	DII
2	Hemmat A et al. (20)	2025	Iran	Retrospective case-control study	80	80	28.00 ± 5.20	36.00 ± 6.70	26.94 ± 3.45	27.34 ± 4.02	3.85 ± 8.56	4.68 ± 8.03	Rotterdam Standard	DII
3	Vaziri Esfarjani F et al. (10)	2025	Iran	Retrospective case-control study	100	100	30.36 ± 5.53	32.24 ± 5.06	29.57 ± 5.23	25.65 ± 4.84	33.90 ± 5.30	38.90 ± 6.34	Rotterdam Standard	DII
4	Wang Q et al. (18)	2022	China	Retrospective case-control study	202	325	30.15 ± 3.40	31.77 ± 3.76	/	/	10.05 ± 11.17	9.80 ± 11.30	Rotterdam Standard	DII
5	Zengin FH et al. (9)	2024	Turkey	Retrospective case-control study	44	44	25.07 ± 1.60	26.49 ± 1.82	27.30 ± 5.10	25.80 ± 5.60	/	/	Rotterdam Standard	DII
6	Zirak Sharkesh E et al. (21)	2022	Iran	Retrospective case-control study	203	291	28.98 ± 5.43	30.15 ± 6.21	25.74 ± 5.44	23.65 ± 3.90	27.32 ± 9.55	33.28 ± 20.97	Rotterdam Standard	DII

### 3.3 Meta-analysis results

**Figure 2:**
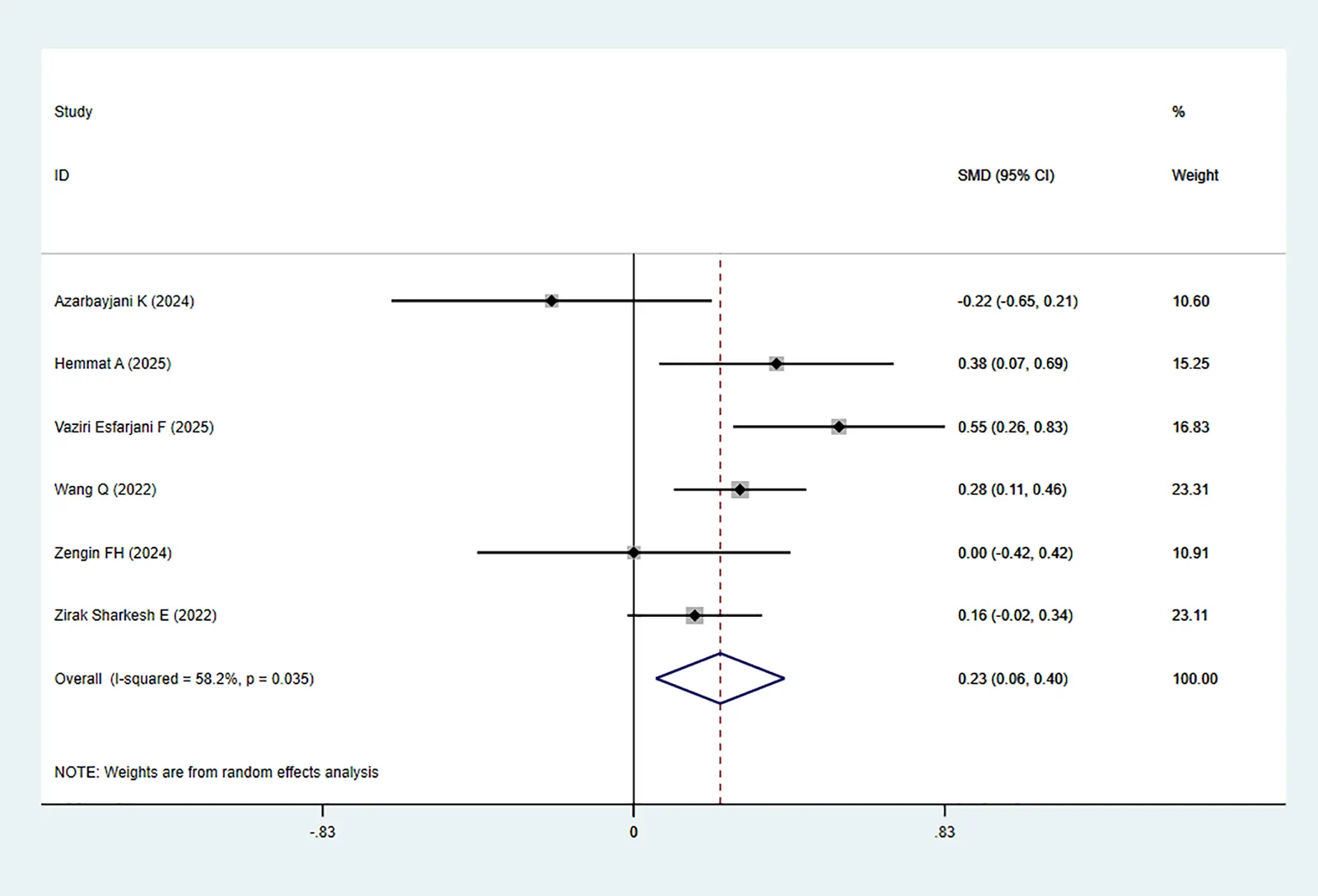
Forest plot for DII levels.

The impact of DII on PCOS status was reported in 6 studies, which exhibited moderate heterogeneity (I^2^ = 58.2%, P = 0.035). A random-effects model was adopted as the default method for pooling effect sizes. Results revealed that the DII levels in healthy participants were slightly but statistically significantly lower than those in PCOS patients (SMD: 0.23; 95% CI: 0.06, 0.40; P = 0.008) (**Figure 2**).

**Figure 3:**
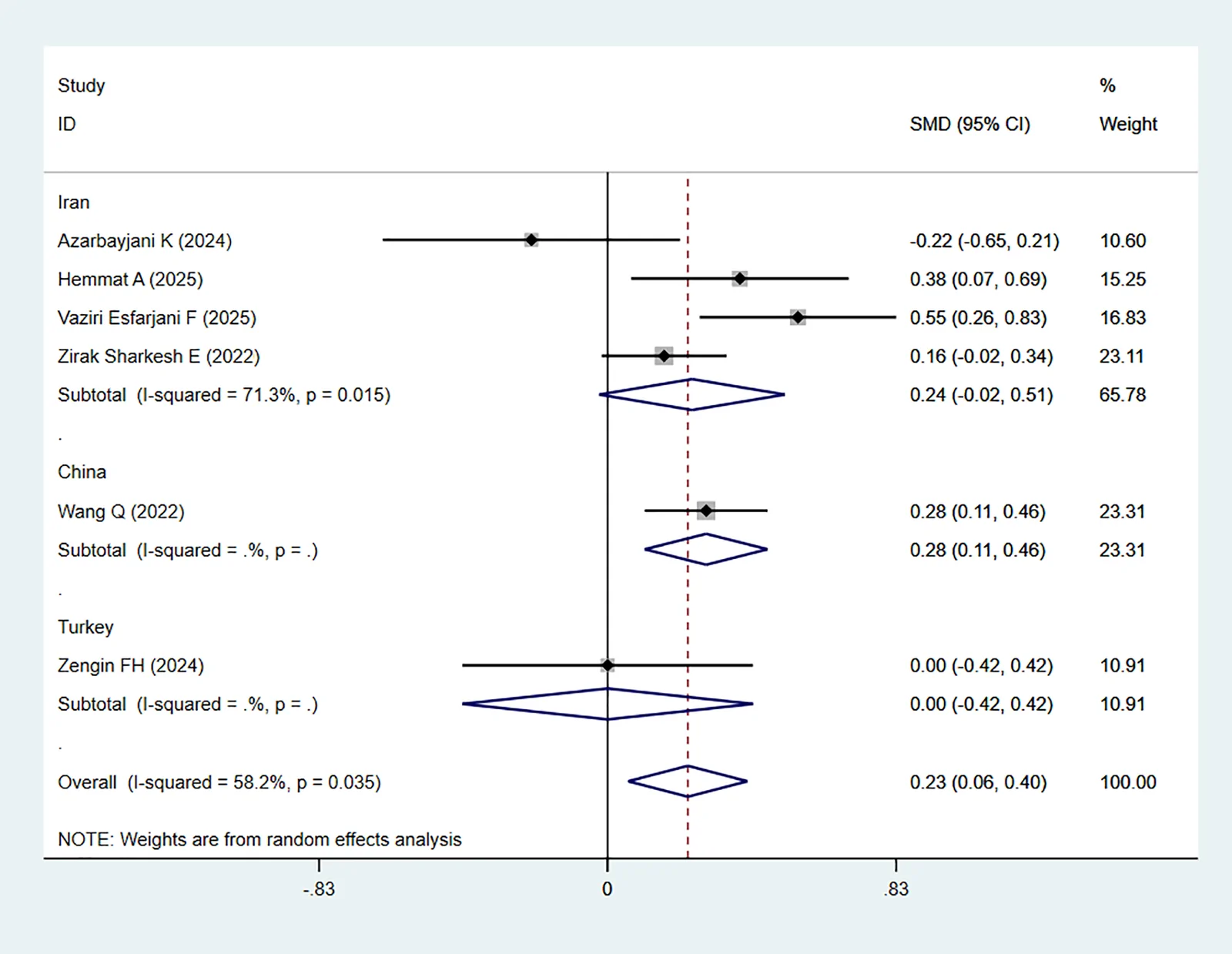
Forest plot for territory groups. There was only one study each in the China and Turkey subgroups; the results were descriptive only, and no inferential conclusions were drawn.

Subgroup analysis by country (**Figure 3**) was performed to identify potential sources of heterogeneity and the impact of different study characteristics on the outcomes. There was only one study each in the China and Turkey subgroups; the results were descriptive only, and no inferential conclusions were drawn.

### 3.4 Sensitivity analysis

**Figure 4:**
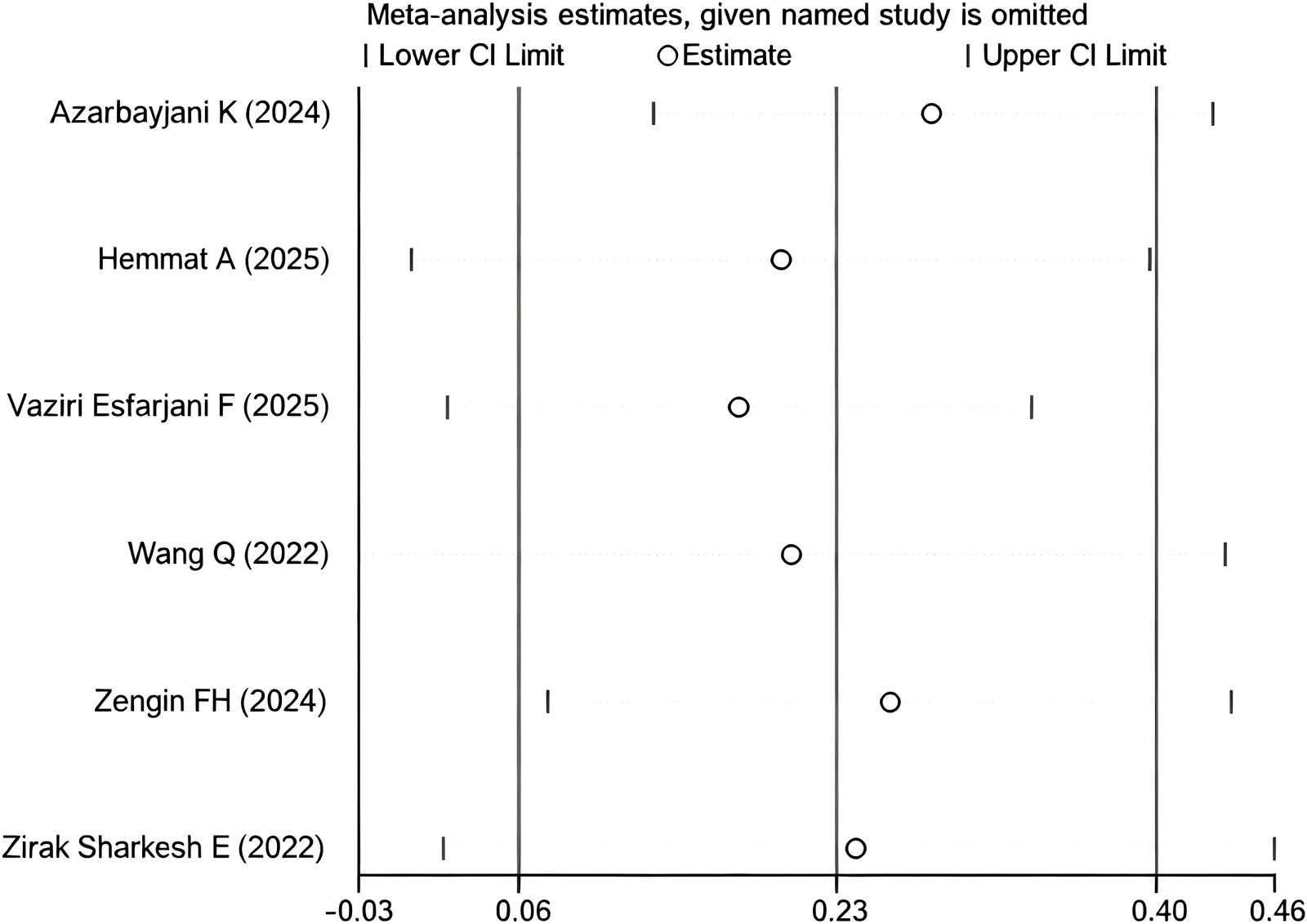
Sensitivity analysis of DII levels.

Sensitivity analysis conducted via the leave-one-out approach failed to detect any marked changes in the pooled effect sizes, suggesting robust study results (**Figure 4**).

### 3.5 Publication bias

Funnel plots (**Supplementary Figure 1**) and Egger’s test showed no statistical evidence of publication bias (P = 0.611); however, with only six studies the statistical power was very low, so publication bias cannot be excluded, and this analysis should be regarded as exploratory (**Supplementary Figures 2**, **3**).

## 4 Discussion

This study represents the first meta-analysis to comprehensively examine the association of DII with PCOS status. It included 6 high-quality case-control studies involving 1,554 women of reproductive age. The results showed modestly higher DII scores in participants with PCOS than in healthy participants, demonstrating a modest association between DII and PCOS. The moderate heterogeneity across studies (I^2^ = 58.2%) may be attributable to differences in factors such as the range of BMI, the phenotypic distribution of PCOS, dietary assessment tools, and dietary patterns across regions. These findings indicate that a highly pro-inflammatory dietary pattern may be a potential risk factor for PCOS, which provides useful evidence-based support for dietary interventions in PCOS management. Given the observational case-control design of all studies, the small number of included studies (k = 6), moderate heterogeneity across studies, and the lack of dose-response relationship analysis and adjustment for key confounders, we rated the certainty of evidence regarding the association between DII and PCOS status as very low.

These findings aligned with prior studies. Existing evidence has indicated that anti-inflammatory dietary patterns, represented by the Mediterranean diet, contain rich omega-3 fatty acids, antioxidants, and dietary fiber, which exert protective effects against PCOS by modulating inflammatory pathways. This finding further suggests that a highly pro-inflammatory dietary pattern may represent a predictor for PCOS and its clinical severity (22). Gautam et al. (23) have demonstrated that anti-inflammatory dietary patterns featuring a low glycemic index, abundant fiber, and high concentrations of omega-3 fatty acids and antioxidants contribute to favorable metabolic and reproductive outcomes in patients with PCOS. Sharkesh et al. (12) have suggested that inflammatory indices may link diet to PCOS.

Pro-inflammatory diets characterized by high DII may represent a major risk factor for PCOS. The underlying mechanism may lie in the fact that this pattern increases intestinal permeability, facilitating the translocation of endotoxins into the bloodstream, which subsequently activates the innate immune inflammatory pathway centered on the TLR4 receptor (11). The resulting systemic chronic low-grade inflammation directly disrupts the ovarian microenvironment, impeding follicular development and normal synthesis of sex hormones, ultimately resulting in the initiation and progression of PCOS (24). A high proportion of refined carbohydrates and high-fat foods in diets provides a greater pro-inflammatory potential, amplifying the association between the DII and PCOS (25).

Subgroup analysis revealed significant heterogeneity among studies conducted in Iran (I^2^ = 71.3%), which may be attributed to differences in the BMI range of the study populations, the composition of the clinical phenotypes of PCOS, dietary assessment tools, and regional dietary patterns. For instance, the difference in the mean BMI of PCOS patients between the studies by Azarbayjani et al. (18) and Sharkesh et al. (20) was approximately 7 kg/m^2^. Obesity itself can enhance inflammatory responses, thereby influencing the strength of the association between DII and PCOS.

This study has several strengths. First, it is the first meta-analysis to synthesize evidence on the association between the DII and PCOS. A comprehensive literature search was performed, and study screening and data extraction were independently conducted by two reviewers. Furthermore, the study protocol was registered in advance (on PROSPERO). Sensitivity analysis further validated the robustness of the findings.

However, this study has several limitations. First, all included studies adopt case-control designs and fall into the category of observational studies, which cannot establish causality and have lower overall quality than randomized controlled trials. Second, this review only reveals a modest association between pro-inflammatory diets and PCOS and fails to verify whether such diets directly increase the probability of developing the disease. Third, the analysis includes only six studies, most of which originate from Middle Eastern countries, limiting the generalizability of the findings to other populations. Fourth, the included studies fail to fully explore the interactions among the components of anti-inflammatory diets and lack stratified analyses for different PCOS phenotypes; therefore, they could not elucidate differences in this association across different phenotypes. Furthermore, due to the small number of included studies, the Egger’s test results have limited reliability, and thus publication bias cannot be ruled out. Given that only six studies were included, the meta-regression lacks statistical power, has a low degree of freedom, and is prone to Type I and Type II errors. Therefore, the failure to detect a significant moderating effect does not imply the absence of such an effect, and the interpretation of the results needs to be validated in a larger sample size. Furthermore, language restrictions may have led to some publication bias. Finally, due to incomplete information reported in the included studies, it is impossible to quantitatively assess residual confounding factors (such as obesity, insulin resistance, total calorie intake, and physical activity levels) that may independently influence DII scores and PCOS status.

The clinical relevance of the observed small effect size warrants consideration. Although statistically significant, the difference in DII scores between PCOS patients and controls is small, and a statistically significant association does not necessarily imply practical clinical relevance. Further studies are needed to replicate and validate the relationship between DII and PCOS. Nevertheless, the DII may serve as a component of an extensive dietary assessment strategy, particularly when combined with other risk factors such as BMI, physical activity, and metabolic parameters. Future research should aim to identify clinically meaningful thresholds for the DII in the risk stratification of PCOS.

Given these limitations, future large-scale, multicenter cohort studies are warranted to establish the causality between DII and PCOS. These studies should expand the geographic scope to include populations of diverse races and dietary patterns to validate the generalizability of this association. Meanwhile, phenotyping PCOS and incorporating confounding factors like dietary component interactions and lifestyle are important to further elucidate the specific mechanisms by which DII influences the pathogenesis of PCOS. Furthermore, future intervention studies may investigate the effectiveness of anti-inflammatory dietary patterns in optimizing inflammatory markers and improving reproductive and metabolic functions in PCOS patients. These studies may provide further direct evidence for developing actionable dietary management strategies for patients with PCOS in clinical practice.

## 5 Conclusion

This meta-analysis revealed a modest association between pro-inflammatory dietary patterns and PCOS status. Although current evidence is primarily derived from observational studies, the findings support the DII as a potential tool for estimating PCOS status associated with diet. Therefore, this study provides preliminary scientific support for considering anti-inflammatory dietary patterns in the management of PCOS. However, statistically significant findings do not equate to practical clinical relevance. The findings of this analysis have only limited reference value and cannot be directly translated into clinical application. Further high-quality prospective studies are required to establish their clinical utility. Future high-quality, multicenter, prospective investigations should be conducted to establish the causality between diet and PCOS and clarify the underlying mechanisms.

## Data Availability

The original contributions presented in the study are included in the article/**Supplementary Material**. Further inquiries can be directed to the corresponding author.
