# Supplementary material for: Association between dietary inflammatory index and polycystic ovary syndrome: a meta-analysis of case-control studies
Source: Front Endocrinol (Lausanne). 2026 Aug 18;17:1843644. doi: 10.3389/fendo.2026.1843644 (PMC13529463; doi:10.3389/fendo.2026.1843644)
Supplement: Supplementary file 2 [file DataSheet1.docx]

Supplementary Table 2 Search Results

| Pubmed | | |
| --- | --- | --- |
| # | Query | Results |
| 1 | "Polycystic Ovary Syndrome"[Mesh] | 20593 |
| 2 | ("cystic ovary"[Title/Abstract] OR "micropolycystic ovary"[Title/Abstract] OR "multiple follicle cyst"[Title/Abstract] OR "ovary polycystic disease"[Title/Abstract] OR "ovary polycystic | 28341 |
| 3 | "Dietary Inflammatory Index"[Title/Abstract] OR "DII"[Title/Abstract] | 5451 |
| 4 | (("cystic ovary"[Title/Abstract] OR "micropolycystic ovary"[Title/Abstract] OR "multiple follicle cyst"[Title/Abstract] OR "ovary polycystic disease"[Title/Abstract] OR "ovary polycystic | 9 |

| Embase | | |
| --- | --- | --- |
| # | Query | Results |
| 1 | 'ovary polycystic disease'/exp | 46023 |
| 2 | 'cystic ovary':ti,ab,kw OR 'micropolycystic ovary':ti,ab,kw OR 'multiple follicle cyst':ti,ab,kw OR 'ovary polycystic disease':ti,ab,kw OR 'ovary polycystic syndrome':ti,ab,kw OR 'polycystic ovarian disease':ti,ab,kw OR 'polycystic ovarian syndrome':ti,ab,kw OR 'polycystic ovary':ti,ab,kw OR 'sclerocystic ovarian degeneration':ti,ab,kw OR 'sclerocystic ovaries':ti,ab,kw OR 'sclerocystic ovary':ti,ab,kw OR 'stein leventhal disease':ti,ab,kw OR 'stein leventhal syndrome':ti,ab,kw OR 'syndrome stein leventhal':ti,ab,kw | 37770 |
| 3 | #1 OR #2 | 49579 |
| 4 | 'dietary inflammatory index':ti,ab,kw OR 'dii':ti,ab,kw | 6737 |
| 5 | #3 AND #4 | 12 |

| Cochrane Library | | |
| --- | --- | --- |
| # | Query | Results |
| 1 | MeSH descriptor: [Polycystic Ovary Syndrome] explode all trees 2199 | 2199 |
| 2 | (‘cystic ovary’ OR ‘micropolycystic ovary’ OR ‘multiple follicle cyst’ OR ‘ovary polycystic disease’ OR ‘ovary polycystic syndrome’ OR ‘polycystic ovarian disease’ OR ‘Polycystic Ovarian Syndrome’ OR ‘polycystic ovary’ OR ‘Sclerocystic Ovarian Degeneration’ OR ‘Sclerocystic Ovaries’ OR ‘Sclerocystic Ovary’ OR ‘stein leventhal disease’ OR ‘Stein Leventhal syndrome’ OR ‘syndrome stein leventhal’):ti,ab,kw | 5668 |
| 3 | #1 OR #2 | 5668 |
| 4 | (‘Dietary Inflammatory Index’ OR ‘DII’):ti,ab,kw | 1639 |
| 5 | #3 AND #4 21 | 21 |
|  |  |  |

| Web of Science | |
| --- | --- |
| Query | Results |
| (cystic ovary) OR (micropolycystic ovary) OR (multiple follicle cyst) OR (ovary polycystic disease) OR (ovary polycystic syndrome) OR (polycystic ovarian disease) OR (Polycystic Ovarian | 42 |

Supplementary Table 3 Newcastle-Ottawa Scale

| ID | study | Selection of study subjects | | | | | Comparability between groups | Measurement of exposure factors | | | Total score |  |
| --- | --- | --- | --- | --- | --- | --- | --- | --- | --- | --- | --- | --- |
|  |  | Representativeness of the exposed cohort | Appropriateness of case definition | Representativeness of the cases | Selection of Controls | Definition of Controls | Consideration of comparability between cases and controls in study design and statistical analysis | Ascertainment of exposure factors | Ascertainment of exposure factors for case and control groups with identical methods | Non-response rate |  |  |
| 1 | Azarbayjani K 2024 | 1 | 1 | 1 | 1 | 1 | 1 | 1 | 1 | 0 | 8 | Good |
| 2 | Hemmat A 2025 | 1 | 1 | 1 | 1 | 1 | 1 | 1 | 1 | 1 | 9 | Good |
| 3 | Sharkesh EZ 2023 | 1 | 1 | 1 | 1 | 1 | 2 | 1 | 1 | 0 | 9 | Good |
| 4 | Vaziri Esfarjani F 2025 | 1 | 1 | 1 | 1 | 1 | 2 | 1 | 1 | 1 | 10 | Good |
| 5 | Wang Q 2022 | 1 | 1 | 1 | 1 | 1 | 2 | 1 | 1 | 0 | 9 | Good |
| 6 | Zengin FH 2024 | 1 | 1 | 1 | 1 | 1 | 1 | 1 | 1 | 1 | 9 | Good |
| 7 | Zirak Sharkesh E 2022 | 1 | 1 | 0 | 1 | 1 | 2 | 1 | 1 | 0 | 8 | Good |

Supplementary Figure 1 Funnel plots

Supplementary Figure 2 Egger's test plot

Supplementary Figure 3 Egger's test results for publication bias
